# Fractures in the Vicinity of the Elbow-Joint

**Published:** 1906-06-23

**Authors:** Andrew Fullerton

**Affiliations:** Hon. Assistant-Surgeon, Royal Victoria Hospital, Belfast, and Belfast Hospital for Sick Children, late Examiner in Surgery, Royal College of Surgeons in Ireland.


### Hospital Clinics.

### FRACTURES IN THE VICINITY OF THE ELBOW-JOINT.

By Andrew Fullerton, M.D., F.R.C.S., Hon. Assistant-Surgeon, Royal Victoria Hospital, Belfast, and Belfast Hospital for Sick Children, late Examiner in Surgery, Royal College of Surgeons in Ireland.

Fractures about the elbow-joint are exceedingly common and are important because of their liability to be followed by serious interference with the movements of the joint. In children, disjunction occurs, but fractures are common at all ages.

The following are frequently met with: (1) Disjunction of lower epiphysis of humerus; (2) transverse supra-condylar fracture; (3) T- or Y-shaped fractures into the elbow-joint; (4) fractures of external condyle and epicondyle; (5) fractures of internal condyle and epicondyle; (6) fracture of olecranon; (7) fracture of coronoid process of ulna; (8) fractures of head and neck of radius.

To investigate a case of injury at the elbow-joint so as to arrive at a correct diagnosis it is important to proceed in a systematic manner. If the patient's forearm is flexed to a right angle, and the relative positions of the three important bony points are noted, it will be found that they form the three angles of a triangle and that the distance between the tip of the olecranon and the external epicondyle is somewhat greater than that between the same point and the internal epicondyle. Also the tip of the olecranon in this position is just anterior to the posterior surface of the upper arm, so that a ruler placed along this surface just misses it. If the forearm is extended the three bony joints are almost on a level, the tip of the olecranon being just above a line drawn through the epicondyles. These points ought to be compared with those of the opposite side and can with more or less difficulty be made out except in cases of excessive swelling, when it becomes difficult or impossible to decide as to the nature of the injury without an anæsthetic or the *x*-rays. If in the flexed position the tip of the olecranon projects behind a line continued from the posterior surface of the upper arm there is either a fracture or disjunction with backward displacement of the lower fragment, or a dislocation of the ulna backwards (fracture of the olecranon with great separation of the fragments will be recognised by other signs). By grasping the lower end of the humerus and attempting to move it on the shaft, crepitus may be felt and will indicate fracture of the lower end of the humerus or disjunction of the lower epiphysis. In dislocation, the relative positions of the three bony points will be disturbed. Each condyle in turn should now be seized and moved if possible on the humerus. It will then be ascertained whether one or both condyles are broken off. The internal epicondyle has an epiphysis in which a nucleus of bone appears about the fifth year. This is often disjoined, and to ascertain the occurrence of this accident, place the finger on the internal condyle when a small flat surface will often be felt, and lower down and somewhat in front a small pea-shaped nodule of bone—the epiphysis—will be found. The thumb should now be placed just below the outer condyle during rotation of the forearm, when the head of the radius may be felt revolving in the orbicular ligament. I should like to draw your attention to the fact that although you may feel this rotation, still there may be a fracture of a portion of the circumference of the head, as in two cases recently under our observation. Crepitus may be difficult to elicit, first on account of pain, and secondly on account of a small fractured segment being driven round by the intact larger portion. Fractures of the head of the radius are more common than one would be led to suppose from the accounts given in text-books and always, unless specially treated, give rise to restricted or totally abolished power of pronation and supination. Therefore, if a patient complains of pain on pressure over the position of the head of the radius, the result of an accident, do not decide too hastily against the possibility of a fracture; you may have to rotate the forearm to the extreme limits of pronation and supination before eliciting crepitus. If still dissatisfied, you must, if possible, have recourse to the *x*-rays. And here I might just say that you ought to cultivate your powers of diagnosis to such an extent that you may have a very fair idea of what is amiss and call the aid of the *x*-rays not to *make* a diagnosis but rather to *confirm* it. The *x*-rays have, I fear, made students careless in diagnosis. It is so easy to see with the rays! Well, you cannot carry the rays about with you, and you will be compelled in private practice in the large majority of cases to do without their aid. It is much better to begin to cultivate your powers of observation now when you are students than when you are practitioners with more limited opportunities. To continue your examination of the joint, measure the distance between the tips of the internal and external epicondyles. This can be done by means of a pair of callipers, or it can be approximately judged by the eye with the fingers placed on the most prominent points. If there is much swelling a tape line measure gives an erroneous idea of the distance, because the line so obtained is not straight but a segment of a circle drawn across the swollen tissues. An increase in breadth indicates a fracture or disjunction with separation of the condyles, and this increase is most marked in T- or Y-shaped fractures into the joint. Now carefully examine the olecranon to see if it moves on the ulna or is separated, more or less widely, from it. If there is much swelling of the part a fissure between the olecranon and ulna may be overlooked, as happened in two cases recently under observation. In this connection I would caution you against the error of diagnosing a fracture from a skiagraph which shows a fissure posteriorly in the olecranon in an adolescent. It is the remains of the cartilage between the epiphysis and the diaphysis, and is a normal condition. Another measurement that should be made in suspected fractures in this region is that from the tubercle always present at the posterior border of the acromion to the external epicondyle. This may help to eliminate dislocation in which the measurements are unaltered unless, indeed, a fracture complicates the dislocation.

The movements of the joint—flexion, extension, and rotation—ought now to be estimated. Ascertain if any lateral movement is present indicating fracture, dislocation, or disjunction. Now examine for the presence of any alteration in the normal carrying angle. The forearm is slightly abducted normally from the line of the upper arm (about 10° to 15°). Any increase—valgus, or diminution—varus, may be due to slipping up of the external or internal condyle respectively upon the shaft of the humerus. The condition, known as the "gun-stock" deformity, is a permanent fixation in the varus position. It does not seriously interfere with the utility of the arm, but is unsightly and must if possible be prevented.

When a considerable amount of swelling and tension of the parts has occurred, and when the patient is unwilling or unable to permit a proper examination of the joint, an anæsthetic may be required and the opportunity taken of placing the fragments in good position after a diagnosis has been made. The *x*-rays, of course, if available, are invaluable in really doubtful cases. The position of the swelling and its point of maximum intensity, so to speak, will be some indication as to the position of the fracture if such exist.

There are certain points in the development of the lower end of the humerus and the upper ends of the forearm bones to which your attention ought to be directed in view of the frequency of disjunction in this region. The lower end of the humerus is developed from four centres. Up to the age of five years it is cartilaginous except for a centre in the capitellum, which, beginning about the second year, gradually extends inwards so as to form a considerable part of the articular end. About the fifth year the epiphysis for the internal epicondyle appears. This remains as a separate centre and is not joined to the rest of the bone until about the eighteenth year. It forms the marked prominence of the condyle, and is very liable to be separated independent of the remaining portion of the lower end of the humerus. In a case recently seen it complicated dislocation backwards of both bones of the forearm. About the twelfth year a centre appears for the inner part of the trochlear portion of the articular end of the humerus. The last centre to appear is that for the external epicondyle at about the thirteenth or fourteenth year. This centre forms the prominence of the external condyle, which is much less marked than that of the internal. The centres for the capitellum, the trochlear portion, and the external epicondyle, having united together, join the shaft at about the sixteenth or seventeenth year, leaving the internal epicondyle still ununited. Before puberty, the lower end of the humerus is cartilaginous well beyond the line of the joint, consequently the latter is not so liable to be implicated in a disjunction as would be the case later when the osseous tissue of the shaft has encroached upon the lower end of the bone so as to lie within the limits of the synovial membrane. The epiphysis for the internal epicondyle, however, remains outside the joint and a disjunction of it will consequently be of less moment, although great swelling of the joint may accompany it, due to laceration of ligaments. Disjunction of the internal epicondyle is occasionally followed by injury to the ulnar nerve as it lies between the condyle and the olecranon.

Disjunction of the lower epiphysis of the humerus is easily recognised as a rule, the soft character of the crepitus and the age of the patient being important aids in the diagnosis. The displacement is usually backward, but lateral and forward displacements may occur. Occasionally one meets with a thickening of the lower end of the humerus some time after this acident. It is due to the tearing up of the periosteum, which is so common an accompaniment of disjunctions. I have seen it recently in a case diagnosed as a sprain. The epiphyses of the upper ends of the radius and ulna are comparatively unimportant from a surgical point of view. That of the ulna is a mere nodule of bone which appears at the extremity of the olecranon about the tenth year and is joined to the diaphysis at about the sixteenth year. At the fifth year or thereabouts the centre for the head of the radius appears and forms a flat plate which gradually thickens and becomes joined to the shaft at the age of seventeen or eighteen years. Separation of this epiphysis as well as of that of the ulna may occur, but are comparatively infrequent, especially the ulnar. As the greater part of the growth of the upper extremity takes place from the upper epiphysis of the humerus, and the lower epiphyses of the forearm bones, it is not liable to be much interfered with by accidents in this region. The complications most to be feared are stiffness and interference with the movements of the elbow-joint.

*The Transverse Supra-condylar* fracture, although usually unconnected with the joint, may seriously interfere with its movements on account of the development of callus which is so liable to limit the movements of flexion and extension, both coronoid and olecranon fossæ being filled up with deposit. The fracture may be caused by falling upon the hand with the arm flexed, or by violence directed to the point of the elbow from behind. In cases seen at this hospital within the last few weeks both forms have been exemplified. A child aged seven years fell upon the hand and had displacement of the lower fragment backwards. In another case, that of an adolescent, the lower fragment was displaced forwards, no doubt due to violence directed from behind. In these cases fracture, and not disjunction, of the lower epiphysis occurred as shown by skiagraphs.

*T- and Y-shaped* fractures are commonly due to severe direct violence, falling from a height, street accidents, etc. The joint is implicated by a longitudinal fracture into it. There are signs of severe trauma and the fracture is often compound. Crepitus and mobility of the fragments separately and together on the shaft are easily obtained, and great broadening of the lower end of the humerus from side to side is present. These fractures present considerable difficulties to treatment and the results are often disappointing.

*Fracture of the External Condyle* is rather a frequent accident and involves not only the condyle but also the capitellum and occasionally the outer portion of the trochlea. The cause is usually a fall upon the hand with the arm extended or slightly flexed, or a fall upon the elbow. The joint is implicated and intra-articular hæmorrhage takes place. The signs are cubitus valgus, diminished (possibly very slightly) distance between tubercle of acromion and external condyle, mobility of condyle on humerus, crepitus and lateral mobility of forearm on arm, increased distance between external epicondyle and olecranon. Bony union usually takes place with or without deformity, but I have recently seen a case several years after the accident in which union failed, the fragment being united to the humerus by a rather loose fibrous band. The head of the radius maintained its position with regard to the fragment and was consequently drawn somewhat upwards. The patient, a boy about 12, has good use of the arm.

The external epicondyle is occasionally fractured nearly always by direct violence, and a separation of its epiphysis may occur in children, but these accidents are much rarer than the corresponding ones on the inner side.

*Fracture of the Inner Condyle*—a comparatively rare occurrence—is usually caused by direct violence—a blow or fall upon the elbow. The signs are the usual signs of fracture with alteration of the normal carrying angle in the direction of adduction or cubitus varus, which may remain as "gunstock deformity," increased measurement from olecranon to epicondyle, mobility of fragment on humerus with crepitus. The fracture is oblique into the joint and takes off a variable amount of the surface for articulation with the ulna, leading perhaps to dislocation of that bone. Last year I operated on a case in which a spicule of bone pressed on the median nerve, causing paralysis, so that this complication must be remembered as possible.

Fracture of the internal epicondyle is usually a disjunction of epiphysis in young people as I have already mentioned in connection with separation of epiphysis. The cause may be direct violence, or the sudden tension of the strong internal lateral ligament which, rather than yield, tears off the portion of bone to which it is attached. Remember the position of the ulnar nerve in connection with this lesion.

*Fracture of the Olecranon* is a relatively frequent accident and is usually produced by direct violence—as by a fall upon the point of the elbow. It may also occur from muscular action, as in throwing, or from hyperextension, as by a fall upon the hand, the olecranon being jammed against the olecranon fossa and the tip broken off. The most frequent seat of fracture is about the middle where the bone is weakest, but fractures at the tip and at the base may occur. The amount of separation of the fragment from the ulna depends upon the extent of laceration of the periosteum and the tendinous fibres of the triceps. Usually a distinct gap exists, due to the drawing up of the fragment by the triceps, but occasionally the separation is slight. It is most marked, of course, in the flexed position. The joint is usually full of blood, and later inflammatory exudate. The ulnar nerve is liable to injury. Union is by fibrous tissue unless the fragments can be brought into accurate apposition by operation or otherwise. The signs of this lesion, when there is much separation, are obvious, but I have seen the fracture missed entirely when only slight separation was present. In well marked cases the patient is unable to extend his forearm against a slight resisting force. Occasionally dislocation forwards of the ulna occurs as a complication.

Fracture of the coronoid process of the ulna is rare, and is usually associated with backward or lateral dislocation of both bones of the forearm. It is said to have been caused by forcible contraction of the brachialis anticus, but this must be extremely rare. If in reduction of a dislocation of both bones of the forearm the deformity tends to recur, it is probably due to a fracture of this process. Flexion will keep the fragment in position.

*Fractures of the Head of the Radius* may occur alone or in conjunction with other injuries about the elbow joint. As I have already mentioned, they are not very rare. There are three cases now attending hospital, upon two of which I have operated. Of these three cases two are in adult males and one in a female of 50. In the two cases operated on—the woman and one of the adult males—a similar condition of affairs was found—namely, the so-called chisel fracture, a segment being broken off from the head in a line perpendicular to the cup-shaped cavity. In both cases the segment was about a third of the circumference, and involved the inner margin. This small segment was in one case further subdivided into two smaller pieces. Probably the usual condition is fracture of the inner margin of the head of the bone. The cause of this fracture is a fall upon the hand with the forearm somewhat flexed and pronated. The force received by the hand is transmitted to the radius, the head of which is forcibly driven against the capitellum. Whether the inner or outer margin of the disc is fractured is said to depend on the amount of adduction or abduction of the limb at the moment of impact. The signs are pain and localised swelling over the position of the superior radio-ulnar joint, and crepitus may be felt on rotation. It may be necessary to have an *x*-ray photograph taken at different degrees of supination to establish the diagnosis. You will remember the position of the radial and posterior interosseous branches of the musculo-spiral nerve in connection with this fracture.

*The Neck of the Radius* may be fractured as the result of direct or indirect violence either alone or in connection with the fracture of the head just described, or perhaps in combination with backward dislocation of the ulna. The signs are limitation or absence of rotation, crepitus, pain and swelling over the affected part. The posterior interosseous nerve is in danger of being injured.

These fractures remind one of an accident which occurs in children known as "pulled elbow," and although this condition is not a fracture, a few words about it may not be out of place in this connection. It is a subluxation of the head of the radius produced by forced traction, and is usually the result of dragging a child by the hand, as in helping him over obstacles or pulling him up on to the footpath from the roadway. Into the gap between the radial head and the capitellum the orbicular ligament is drawn and nipped. The signs are slight flexion of the elbow, inability to use the forearm, which hangs as if paralysed by the side, tenderness over the head of the radius, and pain on flexion, extension and supination. During the examination the head of the radius is frequently restored with a slightly perceptible snap, and the child immediately proceeds to use the limb.

*Treatment of Fractures in the vicinity of the Elbow Joint.*—Since the introduction of the acutely flexed position in the treatment of the fractures I have just described, the prognosis has greatly improved. Of course each case must be treated on its merits, and no blind following of any general rule, will give good results with any form of treatment, and you must satisfy yourselves, whatever method you use, that the fragments are in as good position as it is possible to obtain. The method is carried out as follows: The forearm is acutely flexed upon the arm, taking care not to obliterate the pulse in the radial artery by too great pressure upon the vessels at the bend of the elbow. This is liable to occur if there is much swelling. The flexion is maintained by means of a band of adhesive plaster about 2½ inches wide carried round arm and forearm, so as to be placed upon the arm just below the axilla and upon the forearm just above the head of the ulna. When the swelling is considerable it will be difficult to secure the required amount of flexion, but in a day or two the former will have sufficiently subsided to allow of the correct position being obtained. A thin layer of cotton-wool well dusted with boracic or zinc powder should be placed in the bend of the elbow to prevent chafing. If much swelling occurs at the elbow below the level of the strapping the latter must be removed and put on more loosely. If the strapping chafes or cuts the skin, as it is liable to do, a layer of gauze may be placed between it and the skin, leaving a portion free to allow of the adhesion of the strapping which is necessary to prevent slipping. A sling is used to support the limb, and the dressing is complete. If the chest wall is chafed by contact with the limb or the strapping it may be powdered, or a layer of lint or cotton-wool may be secured in such a way as to prevent this. This method of treatment is intended to *retain* the fragments in position. It is necessary before applying it to manipulate the fragments into as good position as possible. The *rationale* of the method is easily understood: the sigmoid fossa with the coronoid in front and the olecranon behind, assisted by the triceps and the fasciæ round the joint, bind the fragments snugly into position, and keep them securely applied to the shaft of the humerus. Besides this the coronoid, by occupying its own fossa, prevents the latter from being obliterated by callus, thus maintaining the power of flexion, which is more important than that of extension. The latter may be safely left to take care of itself. The cases most suitable for this treatment are: Fractures of the condyles, T- or Y-shaped fractures, some cases of disjunction of the lower epiphysis of the humerus, and probably fracture of the coronoid process. Fractures or disjunctions of the epicondyles do very well as a rule with an internal angular splint. Passive movement should be begun in a week or 10 days. Supracondylar fracture, and some cases of disjunction of the lower epiphysis, are best treated by an anterior angular splint, with a suitably padded posterior splint to prevent the posterior displacement of the lower fragment, which frequently occurs. If the acutely flexed position is used in these cases care must be taken that the upper end of the lower fragment is not projected backwards. Before beginning passive movement make certain that the fragments are sufficiently united to allow of it, otherwise you will bend or break the newly formed bond of union.

Fractures of the head of the radius are best treated, I believe, by operation and removal of the head of the bone, but I would strongly impress upon you the warning that it is a serious matter to open a joint for the purpose of treating a fracture. The prevention of sepsis presents far more difficulties than appear to be appreciated by the average student—even in his last year—and the result of failure may be the loss of the patient's limb, or even his life. Therefore it may be well to advise that, failing suitable surgical assistance, it will suffice to put up fractures of the head and neck of the radius in the flexed position with the hand midway between pronation and supination, meanwhile intimating to the patient that there is great likelihood of the movements of pronation and supination being diminished or lost. If the detached piece of the head forms a foreign body in the joint it will probably have to be removed sooner or later by operation. With or without operation, early passive movement ought to be undertaken in cases of fracture of the head of the radius. Fracture of the olecranon can in many cases be quite efficiently treated by placing an anterior straight splint on the limb so as to maintain it in a position of almost, but not complete, extension. The latter position is exceedingly irksome, and is not necessary. The fragment may be approximated to the ulna by strapping attached to the splint and hooking over the upper extremity of the olecranon, due care being observed to avoid pressure sores or constriction of the blood-vessels of the limb. If much separation of the fragments occurs, and the patient is an artisan, it will probably be necessary to wire the fragments into position with due aseptic precautions. Good fibrous union is frequently obtained by the first method and bony union by the second.

Some of the other fractures I have described may be better treated by open operation, and, if compound, would no doubt be so treated; but one has great hesitation in recommending indiscriminate wiring, pegging, or screwing of fractures to those, perhaps, whose opportunities for acquiring absolute confidence in their ability to maintain asepsis are not sufficiently great. If you follow the rules I have laid down, and make use of an average amount of common sense, I believe you will be able to treat most of these injuries with satisfaction to yourselves and benefit to your patients.

